# The Anti-Oxidative Role of Micro-Vesicles Derived from Human Wharton-Jelly Mesenchymal Stromal Cells through NOX2/gp91(phox) Suppression in Alleviating Renal Ischemia-Reperfusion Injury in Rats

**DOI:** 10.1371/journal.pone.0092129

**Published:** 2014-03-17

**Authors:** Guangyuan Zhang, Xiangyu Zou, Shuai Miao, Jinjun Chen, Tao Du, Liang Zhong, Guanqun Ju, Guohua Liu, Yingjian Zhu

**Affiliations:** 1 Department of Urology, Shanghai First People's Hospital, School of Medicine, Shanghai Jiao Tong University, Shanghai, China; 2 Shanghai Key Laboratory of Tissue Engineering, Tissue Engineering Center, School of Medicine, Shanghai Jiao Tong University, Shanghai, China; 3 Department of Urology, Henan Provincial People’s Hospital, Zhengzhou, China; 4 Department of Urology, Shanghai Children’s Medical Center, School of Medicine, Shanghai Jiao Tong University, Shanghai, China; University of Torino, Italy

## Abstract

Oxidative stress is known as one of the main contributors in renal ischemia/reperfusion injury (IRI). Here we hypothesized that Micro-vesicles (MVs) derived from human Wharton Jelly mesenchymal stromal cells (hWJMSCs) could protect kidney against IRI through mitigating oxidative stress. MVs isolated from hWJMSCs conditioned medium were injected intravenously in rats immediately after unilateral kidney ischemia for 60 min. The animals were sacrificed at 24h, 48h and 2 weeks respectively after reperfusion. Our results show that the expression of NOX2 and reactive oxygen species (ROS) in injured kidney tissues was declined and the oxidative stress was alleviated in MVs group at 24h and 48h in parallel with the reduced apoptosis and enhanced proliferation of cells. IRI-initiated fibrosis was abrogated by MVs coincident with renal function amelioration at 2 weeks. NOX2 was also found down-regulated by MVs both in human umbilical vein endothelial cells (HUVEC) and NRK-52E cell line under hypoxia injury model *in vitro*. In conclusion, a single administration of hWJMSC-MVs might protect the kidney by alleviation of the oxidative stress in the early stage of kidney IRI through suppressing NOX2 expression. Moreover, it could reduce the fibrosis and improved renal function.

## Introduction

Renal ischemia-reperfusion injury (IRI) is common in several clinical situations including shock, sepsis or during transplantation [1]. Severe IRI induced acute kidney injury (AKI) could trigger renal fibrosis in late stage because of proliferation of fibroblasts and excessive deposition of extracellular matrix [2]. Thus AKI is considered as one of main contributors of end-stage kidney disease which leads to high mortality [3]. Preventive therapeutic strategies for IRI might include ischemic conditioning which involves short, nonlethal episodes of ischemia [4], several metabolic intervention options [5], [6], therapeutic gas application [7], modulation of nucleotide signaling [8], [9] and microRNA application [10]. In recent years, a promising and effective approach of therapeutic strategy for renal IRI is the use of mesenchymal stromal cells (MSCs) derived from various sources, such as bone marrow or adipose. However, the mechanisms are under debate.

It is well known that ROS is the key player and an initiator in IRI pathology process [11]. Reperfusion of ischemic tissues results in oxygen burst which means abundant formation of toxic ROS with at least one unpaired electron, including superoxide anion (O_2_^−^), hydrogen peroxide (H_2_O_2_), hydroxyl ( OH) and so on [12]. These ROS with direct cytotoxic effects could react with nucleic acids, functional proteins and plasma membranes, thereby leading to oxidative stress as well as cell death [13]. The sources of ROS in IRI include mitochondria [14], [15], NO synthase [16], xanthine oxidoreductase (XOR) [17] and NADPH oxidase (NOX). NOX, so far as is known, is unique among these for its sole function of producing ROS [18], [19]. And it has been thought that ROS produced by NOX, rather than mitochondrial ROS, play the key role in the case of cell death in IRI [20]. NOX2, also known as gp91 (phox), is the prototype NADPH oxidase which is first found in phagocytes, and also expressed in renal vessels and renal cortex [21], [22]. It has been reported that the NOX2 protein was up-regulated in IR injury [23]. And NOX2 knocking-out [24] or small-molecule NOX Inhibitors [25] show a protective property against IR injury. These studies indicate that NOX2 might be a target molecular for anti-oxidative therapy of renal IRI.

It’s also demonstrated that MSCs display an anti-oxidative activity in tissue injury induced by various reasons [26]–[29], and the reno-protective role through mitigating oxidative stress by MSCs in kidney IRI was also suggested [30], [31]. The paracrine and endocrine mechanisms of MSCs in tissue injury repair have been attached more importance recently [32], [33]. Micro-vesicles (MVs), which are membranous structures delivering bioactive molecular content include proteins, mRNAs and miRNAs sequences, are described as a novel pathway of cell-to-cell interaction and a crucial point of endocrine [34], [35]. It’s also reported that MVs derived from MSCs deceased oxidative stress in myocardial IRI [36] and cisplatin-induced renal injury [37]. However, the mechanisms are unclear and whether hWJMSC-MVs could mitigate the oxidative stress induced by IRI in kidney is unknown.

In the present study, we investigated the anti-oxidative effects of hWJMSCs- MVs and its therapeutic role in kidney injury triggered by ischemia-reperfusion in rats. Moreover, we tested the hypothesis that the reno-protective role of hWJMSC-MVs may be through the suppression of NOX2.

## Materials and Methods

### Ethics statement

In this study, all research involving human participants was approved by the institutional review board of the Chinese Academy of Medical Science and Medical School of Shanghai Jiao Tong University. Human individuals in this study gave written informed consent to participate in research and allow us to publish the case details. This study was carried out in strict accordance with the recommendations in the Guide for the Care and Use of Laboratory Animals of Shanghai Jiao Tong University. The protocol was approved by the Committee on the Ethics of Animal Experiments of Shanghai Jiao Tong University. All surgery was performed under sodium pentobarbital anesthesia, and all efforts were made to minimize suffering.

### Cell culture

Human Umbilical cords were delivered and stored in cold Hank’s balanced salt solution (Sigma-Aldrich, St Louis, MO) and then cellular isolation stared within 4h. The hWJ-MSCs isolation and identification were performed as described previously (Du et al., 2013). Briefly, after the elimination of umbilical cord vessels, mesenchymal tissues were cut into 1 mm^3^ pieces and then stuck to the substrate of culture plates individually, followed by the addition of Low-glucose Dulbecco, Modified Eagle’s Medium (DMEM, Gibco BRL Co.Ltd., USA) containing 10% fetal bovine serum (FBS, Gibco BRL Co. Ltd., USA) at 37°C in a humidified atmosphere with 5% CO_2_. The medium was changed every two days. After two week culture, the adherent cells were harvested with 0.25% trypsin (Gibco BRL Co. Ltd., USA) treatment and sub-cultured. Only cells from 3 to 6 passages were used for experiments.

Human umbilical vein endothelial cells (HUVEC) were isolated from umbilical cord vein by collagenase digestion. The HUVECs isolation was performed as described previously. In brief, the veins were washed twice with phosphate-buffered saline (PBS), and the HUVECs were flushed out after digestion with 0.1% collagenase (Gibco BRL Co. Ltd., USA) for 30 min at room temperature. HUVECs were seeded onto dishes in EGM-2 medium (Lonza., USA) at 37°C in a humidified atmosphere with 5% CO_2_. The medium was changed every two days. After one week culture, the adherent cells were harvested with 0.05% trypsin (Gibco BRL Co. Ltd., USA) treatment and sub-cultured. Only cells from passage 2 to 4 were used for experiments. Phenotypic analysis of cultured cells was determined by immunofluorescence using CD31(Abcam, Cambridge, UK)and VE-cad (Abcam, Cambridge, UK).

NRK-52E cells were obtained from a commercial source (Shanghai Institutes for Biological Sciences, Shanghai, China), and cultured in Low-glucose Dulbecco, Modified Eagle^’^s Medium (DMEM, Gibco BRL Co. Ltd., USA) containing 5% fetal bovine serum (FBS, Gibco BRL Co. Ltd., USA) and 1.5 g/L of sodium bicarbonate at 37°C in a humidified atmosphere with 5% CO_2_.

### Isolation of MVs

MVs were obtained from supernatants of hWJMSCs as previously described [38]. Briefly, hWJMSCs were cultured in DMEM without of FBS and add 0.5% bovine serum album (BSA) (Sigma-Aldrich, St Louis, MO) overnight. The viability of the cells cultured overnight was >99% as detected by trypan blue exclusion and no apoptotic cells were detected by TUNEL assay. The conditioned medium was collected and centrifuged at 2000 g for 20 minutes to remove debris, and then ultracentrifuge at 100,000 g in a SW41 swing rotor (Beckman Coulter, Fullerton, CA) for 1 hour at 4°C. MVs were washed once with serum-free M199 medium (Sigma-Aldrich) containing 25 mM HEPES (PH = 7.4) and submitted to a second ultracentrifugation in the same conditions. MVs were stored at –80°C for the experiments. To quantify the protein content, the Bradford protein assay kit (P0006, Beyotime institute of biotechnology, China) was used. We quantified it indirectly, and 100 μg MVs were achieved from about 5×10^5^ MSCs after serum-free overnight.

### Transimission electron microscopy

MVs were fixed with 2.5% glutaraldehyde in PBS for 2h. After MVs were washed, they were ultracentrifuged and suspended in 100 μl of PBS. A 20 μl of MVs was loaded onto a formvar/carbon-coated grid, negatively stained with 3% aqueous phosphotungstic acid for 1minute and observed by transmission electron microscopy (Hitachi H–7650, Japan).

### MVs surface marker analysis

Flow cytometry was used to characterize the isolated MVs. MVs were incubated for 30 minutes at room temperature, 5 ml of latex beads were added and incubated for another 30 minutes at 4°C, then washed in 0.5% BSA in PBS and incubated with different antibodies (CD9, CD34, CD44, CD45,CD63, CD73 or with appropriate isotype control IgG. After washing, MVs-coated beads were immediately analyzed using a FACS Calibur flow cytometer (Becton Dickinson FACS Calibur).

### Animals

All animals were used in male SD Rats (180–200 g) and housed at a constant temperature and humidity, with a 12:12-h light-dark cycle.

### Animal models of unilateral renal IRI

The rats were anesthetized with sodium pentobarbital (50 mg/kg intraperitoneally) and placed on a warming table to maintain a rectal temperature of 37°C. The left renal pedicle was occluded with a non-traumatic vascular clamp for 60 min, during which time the kidney was kept warm and moist. The clamp was then removed, the kidney was observed for return of blood flow, and the incision was sutured. Shame-treated animals were performed in a similar manner, except that the renal vessels were not clamped. 100 μg MVs in 1 mL vehicle (M199, Gibco BRL Co.Ltd., USA) or 1 ml vehicle only were administered via caudal vein immediately after reperfusion. The animals were separated by different groups according to different therapeutic procedures: 1) sham-operated rats (n  =  6); 2) vehicle-injected IRI rats (n  =  6); MVs-injected IRI rats (n  =  6). The rats were sacrificed at 24 h, 48 h, and 2 weeks respectively (n  =  6 in each group at different time point). Blood and kidney samples were collected and submitted to corresponding examination.

### Renal function

After the rat’s right uninjured kidney was removed on day 12, the blood samples were obtained for measurement of plasma BUN and creatinine. Serum creatinine and BUN were measured by a Beckman Analyzer II (Beckman Instruments, Inc).

### Masson’s trichrome staining

Masson’s trichrome staining was employed to assess the deposition of collagen in renal interstitium. The degree of interstitial fibrosis was scored semi-quantitatively on a 0-to-3 scale (0, no lesion; 1, <33% of parenchyma affected by the lesion; 2, 33% to 67% of parenchyma affected by the lesion; 3, >67% of parenchyma affected by the lesion). The scores were assessed by a blinded observer in 100 random high power fields (HPFs) (magnification ×400) of parenchyma for each rat (n  =  6 rats, each group). Total score was obtained by the addition of all scores, with a maximum score of 300.

### Hematoxylin and Eosin (H & E) staining and histopathology scoring

To detect the injury of kidney, the kidneys were fixed in 4% paraformaldehyde (pH 7.4) and gradually dehydrated, then embedded in paraffin. The samples were cut into 4-μM sections and stained with H&E. Histopathology scoring was applied based on a previous study [39] in a blind fashion. The score was given based on grading of tubular necrosis, loss of brush border, cast formation, and tubular dilatation in 10 randomly chosen, non-overlapping fields (200×) as follows: 0 (none), 1 (≤10%), 2 (11–25%), 3 (26–45%), 4 (46–75%), and 5 (≥76%).

### ROS level analysis

ROS level in kidney was assayed as previously described [40] in which a non-polar compound dihydrodichlorofluorescein diacetate (H2 DCFH-DA), after conversion to a polar derivative by intracellular esterases, can rapidly react with ROS to form the highly fluorescent compound dichlorofluorescein [41], [42]. Briefly, the homogenate was diluted 1∶20 times with ice-cold Locke’s buffer to obtain a concentration of 5 mg tissue/ml. The reaction mixture (1 ml) containing Locke’s buffer (pH 7.4), 0.2 ml homogenate or mitochondria (0.5 mg protein) and 10 ml of DCFH-DA (5 mM) was incubated for 15 min at room temperature to allow the DCFH-DA to be incorporated into any membrane-bound vesicles and the diacetate group cleaved by esterases. After 30 min of further incubation, the conversion of DCFH-DA to the fluorescent product DCF was measured using a spectrofluorimeter with excitation at 484 nm and emission at 530 nm. Background fluorescence (conversion of DCFH-DA in the absence of homogenate) was corrected by the inclusion of parallel blanks.

### Examination of Lipid Peroxide

Malondialdehyde (MDA), a product of reduction in the lipid peroxidation processes [43], was detected using supernatant of the renal cortical homogenate, according to the manufacturer’s protocol (Nanjing Jiancheng Bioengineering Institute, Jiangsu, China).

### Oxyblot detection of protein carbonylation

Renal tissues were homogenized in 10 volumes of homogenization buffer [50 mmol/l Tris/HCl (pH 7.4), 1 mmol/l EDTA, 1%(v/v) Nonidet P40, 150 mmol/l NaCl, 0.25% sodium deoxycholate and 1∶100 dilution of protease inhibitor cocktail (Sigma-Aldrich)]. The protein concentration in the samples was determined by the Bradford assay with BSA as a standard. For detection of protein oxidation, an Oxyblot kit (Millipore) was used. The carbonyl groups in proteins were first derivatized with DNPH (2, 4-dinitrophenylhydrazine) in the presence of 6% (w/v) SDS. The kit used is sensitive to detect as little as 10 fmols of dinitrophenyl residues. To determine the specificity, the oxidized proteins provided by the kit were included as a positive control. Treatment of samples with a control solution served as a negative control for the DNPH treatment. The reaction was stopped after incubation for 15 min at room temperature. Immunoreactive bands were visualized by enhanced chemiluminescence (ECL, Millipore, US).

### Immunohistochemistry

One portion of the renal tissue was fixed in 4% Paraformaldehyde, embedded in paraffin. 5um-thick sections were labeled with rabbit antibody to rat NOX2(dilution 1∶1000; Abcam, Cambridge, UK), rabbit antibody to rat α-SMA(dilution 1∶500; Abcam, Cambridge, UK), rabbit antibody to rat Ki67 (dilution 1∶250; Abcam, Cambridge, UK), goat antibody to rat 8- Hydroxyguanosine (8-OHdG) (dilution 1∶200; Abcam, Cambridge, UK) followed by HRP-conjugated secondary antibody using diaminobenzidine (DAB) reagents as substrate and then counterstained with hematoxylin. Negative control was performed by omitting primary antibodies. Under × 40 magnification, scoring for Ki67-positive cells, was carried out by counting the number of positive tubular cell nuclei in 30 random fields from randomly chosen kidney sections for each animals (n  =  6 rats, each group).

### Immunofluorescence staining for NOX2 expression in HUVECs and NRK52E

HUVECs and NRK-52E injured by hypoxia for 6 h were incubated on chamber slides and exposed to MVs or control medium for 48 hours. Subsequently, the slides were fixed in 4% paraformaldehyde and permeabilized with HEPES-250 Triton X100 buffer (Sigma, St. Louis, MO, USA). Indirect immunofluorescence staining was carried out using rabbit antibody to rat NOX2(dilution 1∶200; Abcam, Cambridge, UK) in blocking buffer at 4°C overnight followed by fluorescein isothiocyanate-conjugated secondary antibody (ZhongShan JinQiao, Beijing, China)at room temperature for 1 hour. The fluorescence was detected by microscope (TE2000, Nikon, Japan) in dark.

### TUNEL assay

Tubular cell apoptosis was assessed by a terminal transferase-mediated dUTP nick-end labeling (TUNEL) assay using an In Situ Cell Death Detection Kit (Roche, Mannheim, Germany). Kidney sections were screened for tubular cells of positive nuclei under ×400 magnification. Apoptotic score was achieved by counting the number of positive nuclei in 30 random fields.

### Western blot

Protein concentration was measured with BCA Protein Assay, 30ug of total protein were electrophoresed on an 8% –10% SDS-PAGE gel and then transferred onto nitrocellulose membranes (Millipore, US). Membranes were blocked in 5% non-fat milk in TBS containing 0.1% Tween 20 for 1h at room temperature, and then incubated each membrane with rabbit antibody to rat NOX2(dilution 1∶1000; Abcam, Cambridge, UK) and β-actin antibody overnight at 4°C. After being washed in PBS, each membrane was incubated for 1–2h with a secondary antibody conjugated by peroxidase at room temperature, detected by ECL regent (Millipore, US). The density of each band was analyzed by image-pro plus 6.0 software.

### Real-Time quantitative PCR analysis

RNA was extracted with the TRIZOL Reagent (Invitrogen, Carlsbad, USA) according to the standard protocol. Total RNA was quantified spectrophotometrically. 5ng of RNA was reverse transcribed with the M-MLV reverse transcriptase kit and Oligo-dT primers (Invitrogen, Carlsbad, USA) for 60 min at 42°C. Real time PCR was performed by Taqman gene expression assays (Applied Bio-Systems, Foster, USA) for detection of gene expression of rat α-SMA or rat β-actin. RT PCR was carried out using the following primers:

Rat α-SMA: Forward 5' CCG AGA TCT CAC CGA CTA CC 3'

Reverse 5' TCC AGA GCG ACA TAG CAC AG 3';

Rat β-actin: Forward 5'-CCT CTA TGC CAA CAC AGT-3'

Reverse 5'-AGC ACC AAT CCA CAC AG-3'.

The threshold cycle (CT) for each gene was determined for each sample. The quantification of the target gene was normalized by β-actin. The relative expression of mRNA expression was calculated by 2^−ΔΔCT^. Triplicates of each sample were performed.

### Statistical analysis

Results from at least three independent experiments are reported as the means ± standard deviation (SD). Statistical analyses were performed using SPSS v19.0. A value of *P*<0.05 was considered to be statistically significant.

## Results

### Isolation and expansion of hWJMSCs

The hWJMSCs were isolated and identified as previously showed [44]. Briefly, the cells were spindle-shaped morphology and adhered to plastic surfaces. With FACS analysis, we found that the expression of MSC markers (CD44, CD73, CD90 and CD105) were positive, whereas the expression of hematopoietic markers (CD45, CD34 and CD14) and an endothelial marker (CD31) were negative (data not shown). In addition, these cells harbored the potential to differentiate toward chondrocytes and osteoblasts (data not shown).

### Isolation and characterization of human umbilical vein endothelial cells (HUVECs)

HUVECs were isolated from umbilical cord vein by collagenase digestion. Immunofluorescence analysis revealed the positive expression of CD31 and VE- Cadherin (date not shown).

### Extraction and characterization of hWJMSC-MVs

The hWJMSC-MVs were isolated and characterized as previously described [38]. They were heterogeneous lipid bilayer vesicles of approximately 30 – 500 nm in diameter, and characterized as cup-shaped or irregular-shaped (data not shown). FACS analysis showed that hWJMSC-MVs were positive for some surface molecules typically expressed by hWJMSCs, such as CD9, CD44, CD63, CD73, and negative for CD34, CD45(data not shown).

### The morphological alleviation of ischemia-reperfusion induced kidney injury by hWJMSC-MVs in early stage

**Figure 1:**
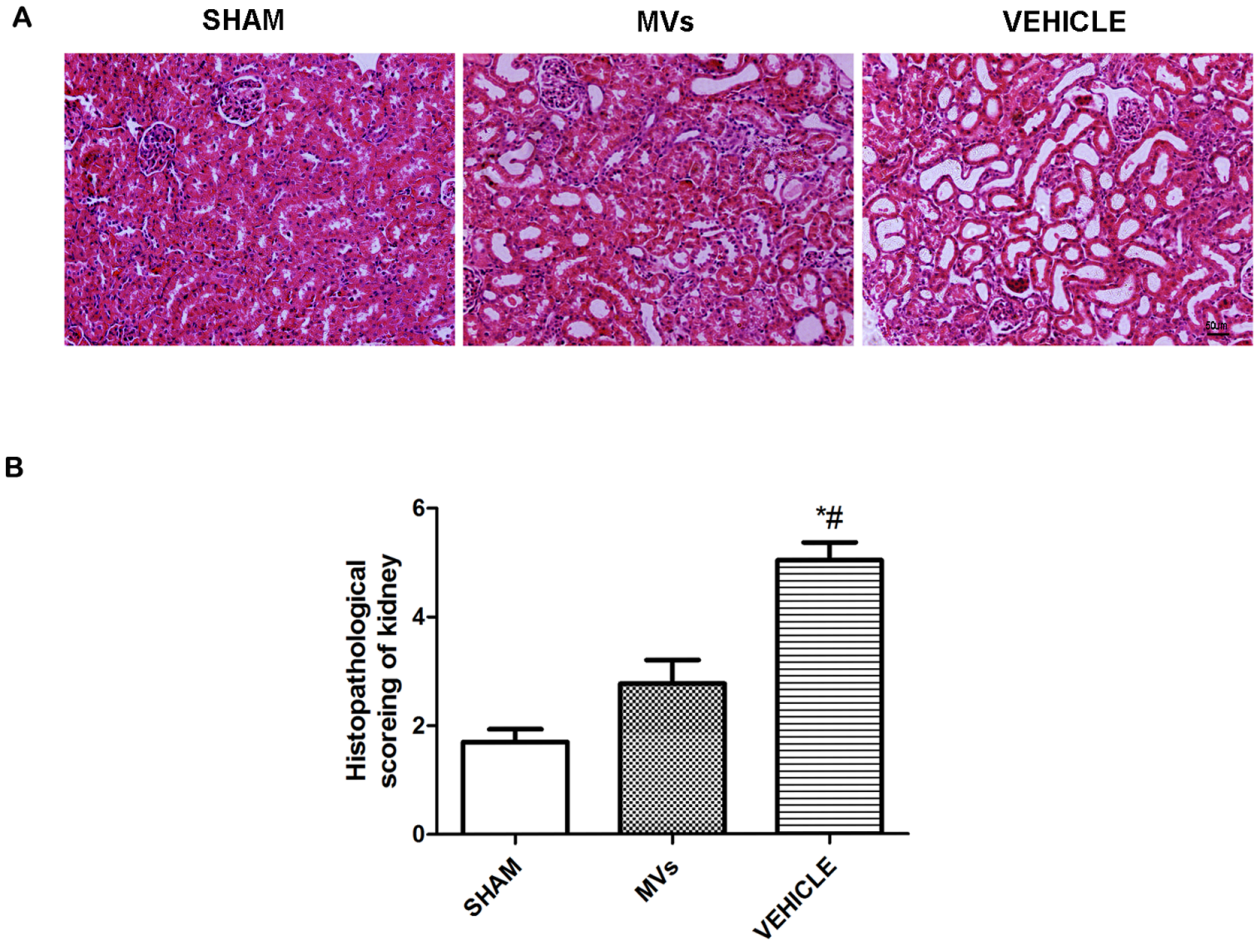
Morphological representation of ischemia-reperfusion induced renal injury in rat at 48h. (A) H & E staining of kidney sections in SHAM operation, MVs and VEHICLE rats, showed notably higher degree cast formation, tubular dilatation and tubular necrosis in VEHICLE group compared with SHAM and MVs treated groups (Original magnification ×200, scale bar  =  50 μm). (B) Histopathological score showed significant difference between SHAM group and VEHICLE group (n = 6, # *P* <0.05), as well as MVs treated group and VEHICLE group (n = 6, *P<0.05).

To evaluate the tubular injury level, H&E staining was performed. Lesions were observed in rats at 48h by H&E staining of kidney tissue slices. The slices showed that numerous necrotic areas of the proximal epithelium had appeared and abundant tubular protein casts had formed in the vehicle and MVs therapy groups. However, the tubular lesions were obviously decreased in the MVs group **(** **Fig. 1A** **).** The representation of histological score suggested significant difference between MV group and VEHICLE group (*P*<0.05). **(** **Fig. 1B** **).**

### The hWJMSC-MVs mitigated the apoptosis and enhanced the proliferation of renal cells

**Figure 2:**
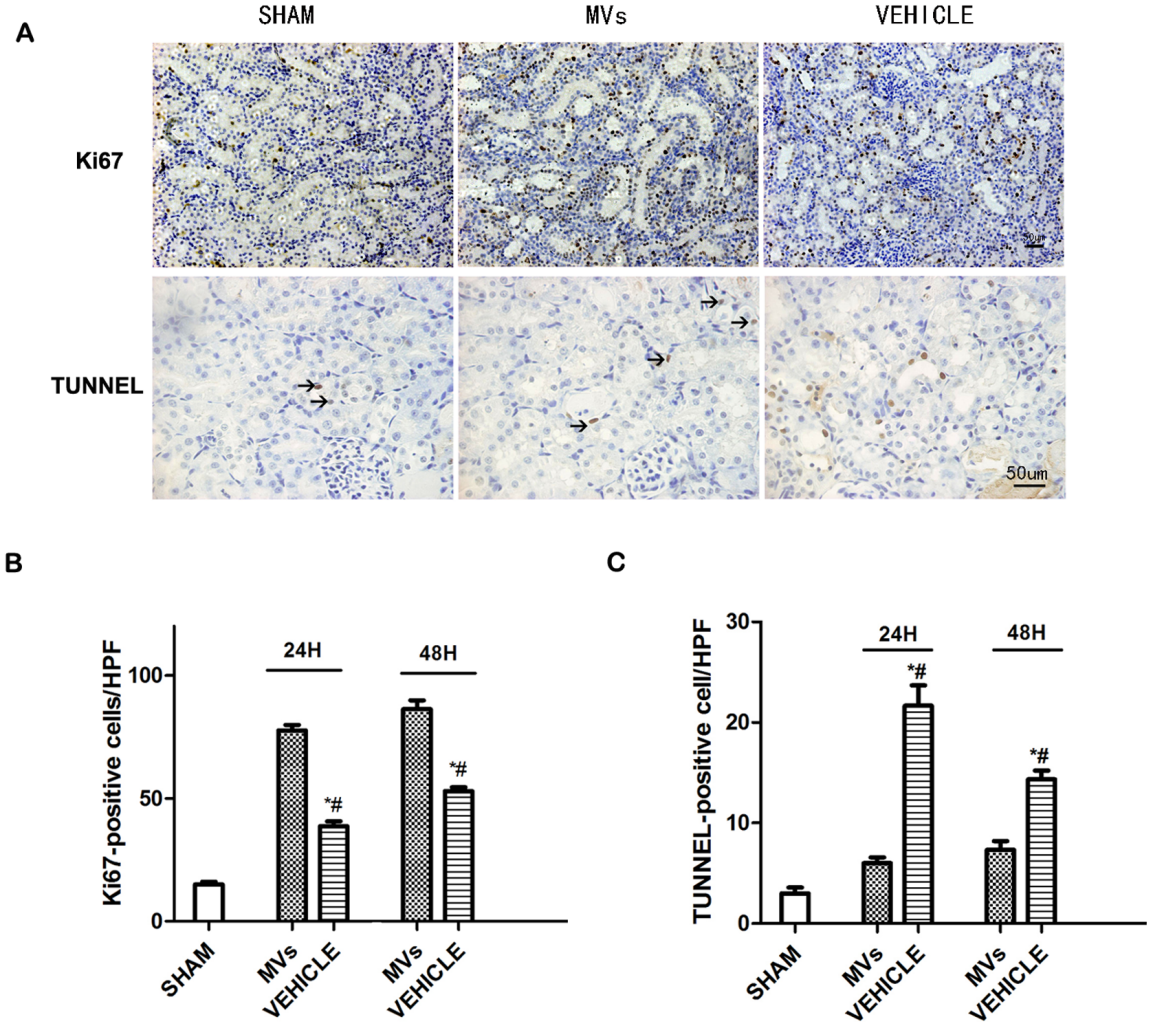
Renal cells apoptosis and proliferation in acute kidney injury rats. (A) Representative micrographs of Ki67 (magnification ×200, Scale bar  =  50µm) and TUNEL (magnification ×400, Scale bar  =  50 μm) immunostaining in the injured kidneys at 48 h after reperfusion. (B) Quantification of TUNEL-positive tubular epithelium cells in the kidney sections at different time points after reperfusion (n  =  6).*P<0.05, MVs versus VEHICLE; #*P*<0.05, SHAM versus VEHICLE. (C) Quantification of Ki67-positive tubular epithelium cells in the kidney sections at different time points after reperfusion (n  =  6). *P<0.05, MVs versus VEHICLE; #*P*<0.05, SHAM versus VEHICLE.

As shown in **Fig. 2A**, the number of TUNEL-positive cells significantly increased at 24h and 48h after IRI compared to the normal group (respectively, 22.5±4.3 versus 2.6±1.0, *P*<0.05 and 14.6±2.0 versus 2.6±1.0, *P*<0.05, **Fig. 2C**), while the kidneys of hWJMSC-MVs treated rats exhibited less TUNEL-positive cells than vehicle group (24h and 48h respectively, 6.9±1.1 versus 22.5±4.3 *P*<0.05, 7.5±1.5 versus 12.8±3.0 *P*<0.05, **Fig. 2C**). Meanwhile, the Ki67 staining (**Fig. 2A**) showed that at 24h and 48h after treated with hWJMSC-MVs the number of proliferating cells increased compared with vehicle treated (respectively, 77.8±6.1versus 38.6±4.7 *P*<0.05, 86.9±7.2 versus 54.3±4.0 *P*<0.05, **Fig. 2B**).

### The ROS level in injured kidney declined as well as oxidative stress mitigated in addition with hWJMSC-MVs in early stage

**Figure 3:**
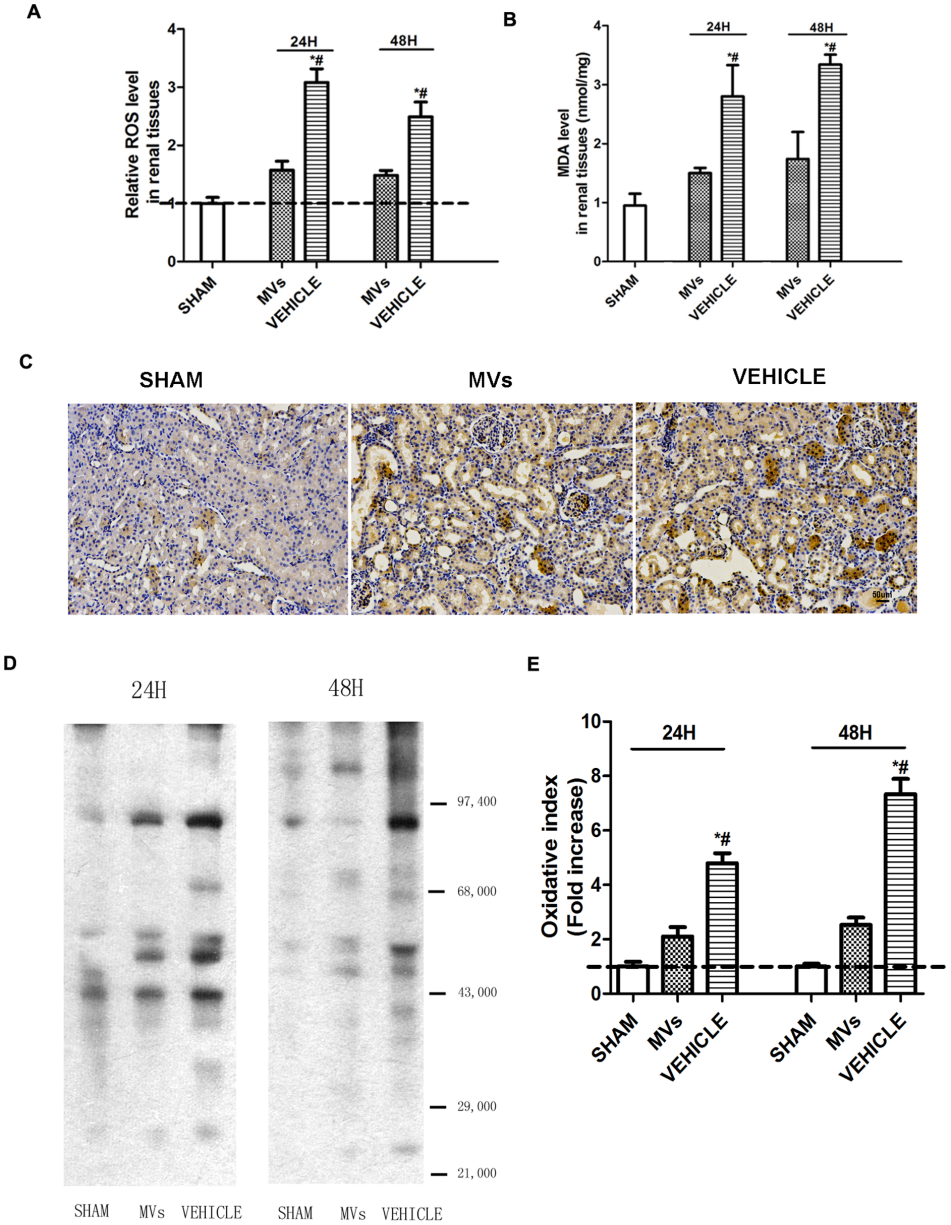
The ROS level and oxidative stress in injured kidney tissues in early stage. (A) DCFH-DA analysis showed relative ROS level in kidney tissues at different time points after reperfusion (n  =  6).*P<0.05, MVs versus VEHICLE; #*P*<0.05, SHAM versus VEHICLE. The ROS level in renal tissues of SHAM-rats was used for calibration (dotted line). (B) Quantification of MDA concentration in renal tissues at different time points after reperfusion (n  = 6).*P<0.05, SHAM versus VEHICLE; #*P*<0.05, MVs versus VEHICLE. (C) Representative micrographs of 8-OHdg immunostaining in SHAM operation, MVs and VEHICLE rats at 48h showed higher staining in the VEHICLE group but reduced by MVs. (magnification ×200, Scale bar  =  50 μm). (D) Oxyblot analysis showed that there was less protein-carbonylation in MVs group compared with VEHICLE group at 24h and 48h after reperfusion. (E) Digital quantification of gray value of oxyblot bands showed the level of injured protein by ROS in each group (n = 3). *P<0.05, MVs versus VEHICLE; #*P*<0.05, SHAM versus VEHICLE. The gray value of SHAM group was used for calibration (dotted line).

In the DCFH-DA analysis at 24h and 48h after reperfusion, elevation of the ROS level in injured kidney tissues as well as obvious reduction in the case of MVs administration were observed, (**Fig. 3A**). As shown in **Fig. 3B**, after ischemia/ reperfusion the MDA level, a classic oxidative biomarker, was significantly elevated in renal tissues, and was reduced by MVs at 24h (vehicle versus MVs, 2.80±0.53 versus 1.50±0.09 μmol/mg, *P*<0.05; vehicle versus sham, 2.80±0.53 versus 0.95±0.20 μ mol/mg, *P*<0.05) and 48h (vehicle versus MVs, 3.34±0.17 versus 1.74±0.46 μmol/mg, *P*<0.05; vehicle versus sham, 3.34±0.17 versus 0.95±0.20 μmol/mg, *P*<0.05) respectively. The 8-OHdg immune-staining which inflects the nucleic acids damaged by ROS was decreased after MVs therapy (**Fig. 3C**). The Oxyblot detection of protein-carbonylation which was shown by **Fig. 3D** and **Fig. 3E** revealed that there was less ROS injured protein level in MVs group compared with the vehicle group.

### IRI-induced fibrosis is abrogated by MVs therapy, as well as improvement of renal function in late stage

**Figure 4:**
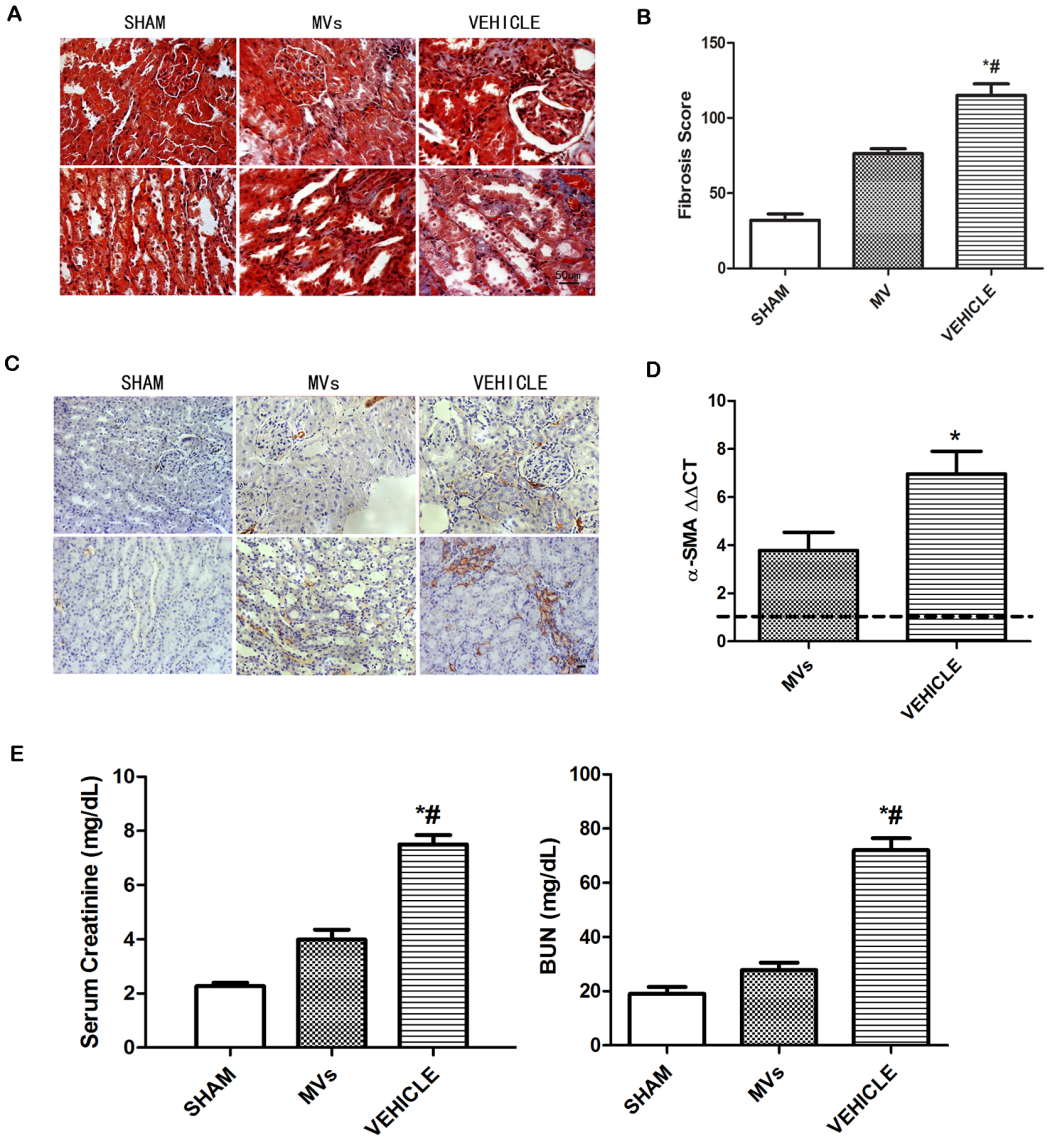
Kidney fibrosis and renal function assessed at 2 weeks after reperfusion in each group. (A) Representative micrographs of Masson’s trichrome staining in tubular interstitial area, ischemic injury caused a progressive increase in positive-staining collagen deposition, whereas MVs group had a remarkable reduction in collagen deposition (The original magnification is ×400, Scale bar  =  50 μm). (B) Fibrosis score was quantification of the Masson’s trichrome staining. The results showed that there was less collagen deposition in MVs group compared with VEHICLE group (n  =  6). *P<0.05, MVs versus VEHICLE; #*P*<0.05, SHAM versus VEHICLE. (C) Representative micrographs of α-SMA immunostaining in SHAM, MVs and VEHICLE rats at 2 weeks. The results revealed that there was higher staining in the VEHICLE group but it was decreased by MVs. (magnification ×200, Scale bar  =  50 μm). (D) The α-SMA mRNA expression in renal tissues of each group by qRT-PCR indicated low expression of α-SMA in MVs group (n  =  6). *P<0.05, MVs versus VEHICLE. The gene expression in SHAM rats was used for calibration (dotted line). (E) Representation of renal function at 2 weeks by BUN and serum creatinine showed strikingly improvement of renal function by MVs therapy (n  =  6). *P<0.05, MVs versus VEHICLE; #*P*<0.05, SHAM versus VEHICLE.

At 2 weeks post-IRI, the exposure to IRI led to the development of significant fibrotic lesions in the renal interstitial area, as indicated by Masson’s trichrome staining. In contrast, after MVs therapy there was only a slight fibrogenesis (**Fig. 4A** and **Fig.4B**). In addition, α-SMA expression on tubular cells, an indicator of epithelial-mesenchymal transition which was shown by IHC, occurred mostly in vehicle-treated animals and slightly in MVs infusion rats (**Fig. 4C**). The α-SMA mRNA detected by qRT-PCR indicated a similar outcome **(** **Fig. 4D** **)**. Furthermore, the level of serum creatinine in animals receiving vehicle infusion was significantly higher compared with that of sham animals and infusion (vehicle versus MVs, 7.50±0.78 versus 3.99±0.81 mg/dl, *P*<0.05; vehicle versus sham, 7.50±0.78 versus 2.27±0.27 mg/dl, *P*<0.05) (Fig. 3C). A similar result was observed when BUN in plasma was assessed (vehicle versus MVs, 72.01±9.98 versus 27.82±6.99 mg/ml, *P*<0.05; vehicle versus sham, 72.01±9.98 versus 19.00±5.70 mg/ml, *P*<0.05) (**Fig. 4E**).

### The down-regulation of NOX2 by MVs both *in vivo* and *in vitro*

**Figure 5:**
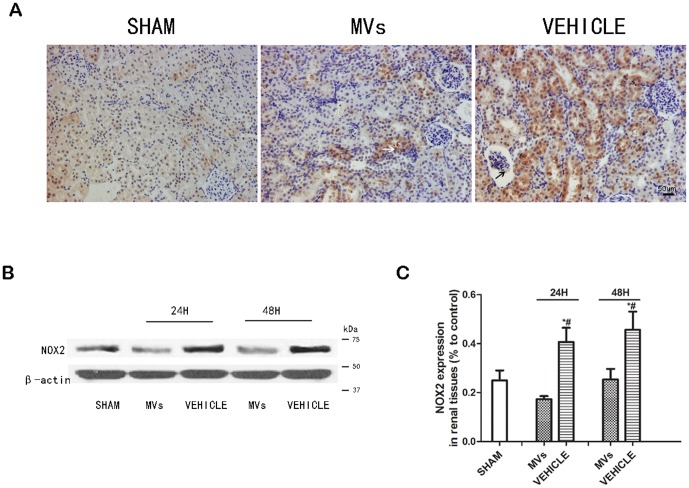
The NOX2 expression in IR induced acute kidney injury in rats. (A) Representative micrographs of NOX2 immunostaining in SHAM operation, MVs and VEHICLE rats at 48h showed down-regulation of NOX2 after MVs treatment. The NOX2 protein was mainly located in renal tubular cells (white arrow), as well as slightly expressed in glomerulus (black arrow). Magnification ×200, scale bar  =  50 μm. (B) Western-blot analysis of NOX2 in renal tissues indicated suppression effects of MVs both at 24 and 48h *in vivo.* (C) Relative quantification of NOX2 expression showed MVs could downregulate NOX2 in IRI kidney (n = 3). *P<0.05, MVs versus VEHICLE; #*P*<0.05, SHAM versus VEHICLE.

**Figure 6:**
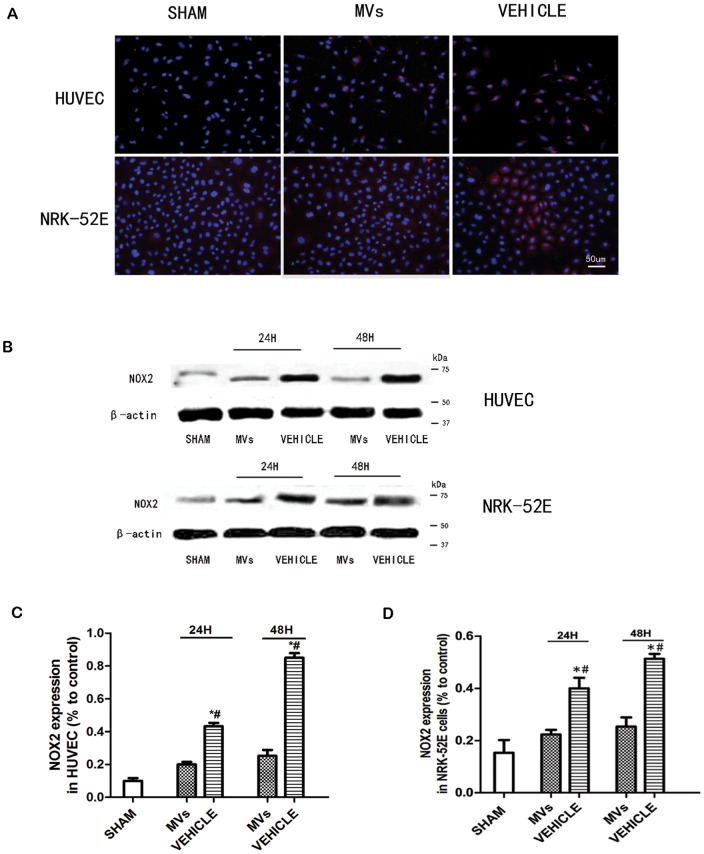
The NOX2 expression in HUVEC and NRK-52E cells under hypoxia model. (A) The Immunofluorescence staining of NOX2 (red) in HUVEC and NRK-52E cells demonstrated that after hypoxia for 6 h there was more fluorescence in VEHICLE group compared with SHAM group, meanwhile MVs treatment group had less NOX2 staining compared with VEHICLE group. The original magnification is ×400, scale bar  =  50 μm. (B) Western-blot analysis showed that there was notably over-expression of NOX2 after injury, and it was strikingly down regulate by MVs both in HUVEC and NRK-52E cells. (C) NOX2 expression by relative gray value represented suppression effects of MVs under hypoxia model. Statistical analysis showed significant difference both in HUVEC and NRK-52E cells between MVs group and VEHICLE group (*P<0.05, MVs versus VEHICLE; #*P*<0.05, SHAM versus VEHICLE).

The suppression effect of MVs on NOX2 was shown by the IHC as well as Western-blot assays. In IHC staining, the NOX2 was found mainly expressed in renal tubular cells especially in injured cells, as well as slightly in glomerulus (**Fig. 5A**). As shown in **Fig. 5B**, the NOX2 protein in injured kidney was up-regulated compared with sham group, but down-regulated at 24h and 48h after MVs intervention. Relative quantification of NOX2 expression showed MVs could down-regulate NOX2 in IRI kidney (*P*<0.05, MVs versus VEHICLE; n = 3 **Fig. 5C**). In order to confirm the effects *in vitro,* we also performed Western-blot and immunofluorescence staining on HUVEC and NRK-52E cells under hypoxia-injured model. The results showed the similar outcomes as the results of *in vivo* (**Fig. 6A-6B**). The western-blot bands grey analysis showed in both of two cell line (**Fig. 6C-6D**) the MVs could down-regulate NOX2, and exhibited significant difference. (*P*<0.05, MVs versus VEHICLE; n = 3).

## Discussion

In the present study, we have observed that a single administration of hWJMSCs-MVs could decrease the ROS level in renal tissue both at 24h and 48h time point after renal IRI. Meanwhile, the MDA evaluation, 8-OHdg staining and Oxyblot analysis showed that the oxidative stress was mitigated by hWJMSCs-MVs. The reduced apoptosis of renal cell was observed as well as enhanced cell proliferation in early stage of IR triggered kidney injury. Furthermore, the renal function was improved and kidney fibrosis was abrogated by MVs at 2 weeks.

In recent years, the strategies of administration of MSCs from various sources such as bone marrow, fetal membrane and adipose in repairing kidney injury are reported [45]–[47], but the potential mechanisms remain unclear. In the previous studies, Caplan et al have suggested that MSCs may provide a paracrine support to kidney repair [48]. Consistently, Bi et al. demonstrated that the administration of conditioned medium from MSCs may also have the beneficial effects of the AKI [33], indicating that endocrine or paracrine mechanism plays an important role. Our group also found that hWJMSCs could alleviate acute and chronic kidney injury through endocrine mechanism [44]. MVs that are contained in the condition medium of MSCs are small vesicles released by cells bearing the surface antigens characteristic of the original cells. It can also enter the target cells through specific receptor ligand interactions and transfer microRNA, mRNA, proteins, and other bioactive materials [49]–[52]. MVs are vesicles composed of exosomes, shedding vesicles and apoptotic bodies. Exosomes range from 30 to 120 nm in size, and their release depends on cytoskeleton activation. While vesicles generated by direct budding of the plasma membrane, also known as shedding vesicles, are more heterogeneous in size and include vesicles ranging from 100 nm to 1 μm in size [53]. In the present study, we isolated MVs from hWJMSCs’ conditioned medium and found that MVs were heterogeneous lipid bilayer vesicles of approximately 30 – 500 nm in diameter, and characterized as cup-shaped or irregular-shaped. We believed that MVs might include not only exosomes, but also shedding vesicles and other apoptotic bodies.

A previous study demonstrated a protective effect of bone marrow MSCs derived MVs on acute kidney injury and found MVs detectable in injured renal tubules at 1h after intravenous administration [52]. In the present study, we found that hWJMSCs-MVs have a crucial therapeutic effect in renal IRI. We believe it has the same efficacy of cell therapy on the functional and morphologic recovery from ischemia reperfusion induced kidney injury in rats. Consistently, Gatti et al showed that MVs derived from bone marrow MSCs protect IRI-induced acute and chronic kidney injury [54], although the mechanism remains unclear.

In order to investigate the potential mechanism, the ROS levels were measured in injured kidney tissues by DCFH-DA analysis which is now the most sensitive method to detect ROS [55]. And we found the ROS level declined at 24h and 48h after MVs therapy, in parallel with the lessened 8-OHdg staining which reflects the nucleic acids damage by ROS. Meanwhile the Oxyblot analysis showed less protein carbonyls detection which means decrease of ROS injured protein in MVs group. Moreover, the renal cell apoptosis was mitigated and proliferation was enhanced in MVs group. Based on the above results, we concluded that MVs derived from hWJ-MSCs could decline the ROS level and thus alleviated the oxidative stress in IR induced kidney injury. Another study also reported that MVs derived MSC decreased oxidative stress in myocardial IRI [36].

To further explore the potential mechanism, we detected the expression of NOX2, the first described NADPH oxidase with a sole function of producing ROS, in IR injured kidney. Interestingly, we found that the NOX2 protein was up-regulated after IRI in renal tissue and was strikingly down-regulated by MVs both at 24h and 48h after reperfusion. A previous study carried out by Cantaluppi V et al. showed that MVs derived from endothelial progenitor cells could be detectable both in endothelia and tubular epithelia cells after intravenous injection in acute kidney injury model [56]. So *in vitro* studies, we also confirmed that MVs could suppress the expression of NOX2 in cultured HUVECs and NRK-52E cell line under hypoxia injury model. In a recent review, NOX was termed as a crucial source of ROS in IRI [19]. Thus we suggest that suppression of NOX2 by MVs might involve in the ROS level decline for its important molecular function. In other words, the low expression of NOX2 presumably account for the less ROS production in MVs group. In addition, previous studies reported that NOX2 knocking-out [24] or small-molecule NOX Inhibitors [25] exhibited protection against IR injury. So we believed that the reno-protective role of hWJMSC-MVs might attribute to NOX2 regulation. Moreover, both of endothelial cells and tubular epithelial cells were regarded as targeted cells of MVs.

In the present study, we also found the ameliorated renal function and abrogated renal fibrosis after 2 week MVs therapy. It is increasingly appreciated that aberrant incomplete repair induced by AKI contributes to chronic fibrotic kidney disease [3]. So we believe that the renal fibrogenesis was abrogated presumably as a result of the amelioration of acute injury repaired by hWJMSC-MVs.

However, there were still some questions in the present study. First, there might be potential bias of cross-reaction between species, as we applied MVs from human to rats. Although, It has been shown that MVs derived from MSCs exhibit low antigenicity following in vivo allogeneic administration [57]. Secondly, how MVs does modulate NOX2 expression is unknown. Some studies showed that MVs derived from various sources could horizontal transfer nuclear acids, functional proteins, bioactive membrane and other materials [58], [59]. In our opinion, any of these could take part in the modulation of NOX2, and the process might be sophisticated. As [56] Cantaluppi et al. also reported microvesicles derived from endothelial progenitor cells protected the kidney from ischemic acute injury by delivering their RNA content. Thus, we preferred that some patterns of microRNAs might be account for the effect as a post-transcription regulation, although other probabilities are not excluded. We plan to investigate the deeper mechanism in the next project.

In conclusion, hWJMSC-MVs therapy exerts strikingly beneficial effects on IR induced kidney injury. In addition, hWJMSC-MVs could alleviate oxidative stress due to suppression of NOX2, which may be a new insight into the mechanisms for the therapeutic effects of hWJMSCs on renal IRI
